# Efficacy and safety of tranexamic acid in geriatric hip fracture with hemiarthroplasty: a retrospective cohort study

**DOI:** 10.1186/s12891-019-2670-5

**Published:** 2019-06-28

**Authors:** Jinwei Xie, Qinsheng Hu, Qiang Huang, Guo Chen, Zongke Zhou, Fuxing Pei

**Affiliations:** Department of Orthopaedic surgery, National Clinical Research Center for Geriatrics, West China Hospital, Sichuan University, 37#Guoxue Road, Chengdu, Sichuan Province 610041 People’s Republic of China

**Keywords:** Tranexamic acid, Hip fracture, Blood management, Fast-track surgery

## Abstract

**Background:**

Geriatric hip fracture patients are particularly susceptible to blood loss and venous thromboembolism (VTE) during hemiarthroplasty, yet relatively few studies have examined the safety and efficacy of tranexamic acid (TXA) in these patients.

**Methods:**

This cohort study of hip fracture patients (≥65 years) undergoing hemiarthroplasty between January 2013 and September 2016 involved 289 patients who received 15 mg/kg TXA prior to surgery and 320 who received no TXA. All patients underwent a fast-track program including nutrition, blood, and pain management; VTE prophylaxis; early mobilization; and early intake. The primary outcome was red cell transfusion requirement. Secondary outcomes included blood loss, hemoglobin (Hb) level, VTE, adverse events and length of hospital stay. Multivariate logistic regression and meta-analysis of the literature were also performed to control for confounding factors and identify risk factors of red cell transfusion.

**Results:**

The proportion of patients receiving at least 1 U of erythrocytes was significantly lower in the TXA group (8.65%) than in the control group (24.06%, OR 0.299, *p* < 0.001). Mean Hb level was significantly higher in the TXA group on postoperative day 1 (111.70 ± 18.40 vs 107.29 ± 18.70 g/L, *p* = 0.008) and postoperative day 3 (108.16 ± 17.25 vs 104.22 ± 15.16 g/L, *p* = 0.005). A significantly higher proportion of TXA patients began to ambulate within 24 h after surgery (37.02% vs 26.25%, *p* = 0.004), and their length of hospitalization was significantly shorter (11.82 ± 4.39 vs 15.96 ± 7.30 days, *p* = 0.003). TXA did not increase risk of DVT (OR 0.70, 95%CI 0.25 to 1.97). Logistic regression showed that, after adjusting for covariates, TXA was associated with 62% lower risk of red blood cell transfusion (0.327, 95%CI 0.214 to 0.696), and a similar result was obtained in meta-analysis of unadjusted data from the present study and the literature (OR 0.33, 95%CI 0.25 to 0.43).

**Conclusion:**

TXA appears to be safe and effective for reducing blood loss and red blood cell transfusion in geriatric hip fracture patients undergoing fast-track hemiarthroplasty.

## Background

In 2000, 1.6 million geriatric patients (≥65 years) worldwide suffered from hip fracture, and this number is 27% higher than in 1990, reflecting population aging [1]; this number is expected to grow to 2.6 million in 2025 and 4.5 million in 2050 [2]. Hip fractures have a major impact on health-related quality of life, and represent a major source of health-care expenditure [3, 4]. Mortality among elderly male patients with hip fracture is as high as 32.5, and 21.9% among female patients at 1 year after fracture [5]; in surviving patients, hip fractures reduce daily life activities and quality of life. Therefore, helping these patients achieve early mobility is critical for recovery.

Hemiarthroplasty is one of the most common surgeries for elderly hip fracture patients. Because of trauma during hip fracture, the combination of hidden blood loss and intraoperative blood loss may be as high as 1301 ml [6, 7]. A substantial proportion of these patients require transfusion; the precise proportion varies from 26.2 to 39.5%, reflecting different transfusion thresholds [8, 9]. A proportion of these patients must receive allogeneic blood transfusion, which can give rise to serious complications such as immunological reaction and disease transmission, and which can increase risk of surgical site infection, increase costs and prolong hospital stay. Substantial anemia can exacerbate comorbidities in these patients. Thus numerous blood-sparing techniques have been developed to reduce the transfusion requirements and enhance recovery after surgery; these techniques include perioperative blood.

Salvage, controlled hypotension anesthesia, antifibrinolytic agents and erythropoiesis - stimulating agents.

A particularly effective intraoperative blood-sparing treatment is tranexamic acid (TXA), a synthetic antifibrinolytic agent, which competitively inhibits plasminogen activation by blocking lysine binding sites, thereby inhibiting the breakdown of clots. This can reduce blood loss and transfusion requirements. Numerous studies over the past decades have investigated the TXA efficacy and safety in patients undergoing elective orthopedic surgery, including total joint arthroplasty and spine surgery [10–12]. The overwhelming consensus from these studies is that regardless of the route of administration, TXA is therapeutically effective, cost-effective and safe [10, 11, 13], although some studies indicate residual questions about safety [14].

Compared to most of the patient populations analyzed in the TXA evidence base, elderly patients with hip fracture often have multiple comorbidities and so are more susceptible to adverse events from blood loss and immobilization. For example, TXA in elderly patients with hip fracture has been associated with greater risk of vascular events at 6 weeks [15], leading those authors to conclude that further safety evaluation is required before off-label TXA use can be recommended. Thus, we conducted a retrospective cohort study in geriatric hip fracture patients older than 65 years with hemiarthroplasty in order to help clarify whether (1) TXA can decrease blood loss and the red blood cell transfusion rate, and whether (2) TXA is safe.

## Methods

### Study design

This retrospective cohort study was conducted from January 2013 to September 2016 in five large joint reconstruction centers within the framework of a National Health Ministry program designed to assess the efficacy and safety of arthroplasty based on data collected from 27 large teaching hospitals in China and stored in a central database. The research program was approved by the Institutional Review Committee of West China Hospital of Sichuan University (2012–268), and all procedures were performed according to the Declaration of Helsinki. All patients received written informed consent before operation.

#### Inclusion and exclusion criteria

Patients were selected from the central database if they were (1) geriatric patients (≥65 years) undergoing primary hemiarthroplasty for hip fracture; (2) with a normal preoperative platelets level and coagulation function; and (3) with negative find of preoperative lower limb Doppler ultrasound. Exclusion criteria were as following: (1) history of deep venous thrombosis (DVT) or pulmonary embolism (PE); (2) history of myocardial infarction or stroke within the previous 3 months; (3) known to be allergic to TXA; (4) severe hepatic or renal insufficiency; or (5) multiple fractures.

#### Enhanced recovery after surgery (ERAS) protocol

The ERAS protocol was similar across the five centers, which included preoperative comorbidity assessment, blood management, pain management, VTE prophylaxis, anesthesia and surgery.

#### Preoperative comorbidity assessment

Cardio-pulmonary function was assessed after admission through various tests. Blood pressure was regulated to remain below 140/90 mmHg, and the level of fasting blood glucose was controlled to be 8–10 mmol/L. Patients were taught breathing exercises to improve pulmonary function.

#### Surgical procedures and anesthesia

All surgeries were performed under general, spinal or combined spinal-epidural anesthesia (CSE), depending on what the anesthesia team considered appropriate for the individual patient. Preoperative oral carbohydrate treatment and intraoperative goal-directed fluid therapy (4% < SVV ≤ 9%) were adapted. All procedures were operated via a posterolateral approach with application of bipolar cementless or cement prosthesis.

#### Blood management protocol

Blood management involved four strategies. First, patients were encouraged to eat food high in protein and vitamins, such as eggs, meats and vegetables. If patients had poor appetite, the nutritionist prescribed enteral nutrition preparations in order to maintain albumin level ≥ 35 g/l. Second, patients diagnosed with anemia (Hb < 130 g/L for men, < 120 g/L for women) received erythropoietin (10,000 U) once daily for 5 days, and intravenous iron (200 mg) every other day. Third, TXA was injected intravenously (15 mg/kg) 10 min prior to incision if deemed necessary by the surgeon, based on standard practices at the participating hospitals. Fourth, controlled hypotensive anesthesia (MAP 50–65 mmHg) was used during surgery.

The decisions on whether red blood cell (RBC) transfusion was necessary or not were made by surgeons during operation, or by attending physicians after operation, according to the criteria for red blood cell transfusion recommended by the Chinese National Ministry of Health: (a) Hb < 70 g/L or (b) 70–100 g/L with symptomatic anemia [16].

#### Pain management protocol

The principal analgesic drugs used during the study were non-steroidal anti-inflammatory drugs; Ropivacaine (Naropin, 20 mg/ml, AstraZeneca, Sweden) was given to all patients intraoperatively by periarticular injection. Sleep and anxiolytic therapies were administered when necessary, especially to patients with insomnia and anxiety.

#### Venous thromboembolism (VTE) prophylaxis

Physical prophylaxis and chemoprophylaxis were taken precautions against VTE, which included quadriceps strength exercises, early ambulation and anticoagulant therapies. Quadriceps strength exercises were achieved by dorsal extension, plantar flexion of ankle joint, and straight leg raising practice since admission. All patients began to walk with partial weight-bearing after review of hip x-ray images on postoperative day 1. Use and timing of anticoagulants varied among the study centers. All patients were followed up for 3 months. Ultrasonography was conducted immediately if DVT was clinically suspected, while enhanced computed tomography was conducted for clinically suspected cases of PE.

### Outcome measurements

All the needed data were extracted from the central database, which included patients’ demographic data, anesthesia data, surgical data, Hb and hematocrit (Hct) level (on admission, on postoperative days 1 and 3), incidence of RBC transfusion and other adverse events.

The primary outcome was RBC transfusion requirement before discharge. And secondary outcomes were Hb level, total blood loss (TBL), length of hospital stay, incidence of DVT, PE and other adverse events.

TBL was calculated using the *Gross* and *Nadel* formula [17, 18] like our previous studies [16]. TBL = PBV × (Hct_pre_ − Hct_post_) / Hct_avg_, where Hct_pre_ refers to preoperative Hct, Hct_post_ to Hct on the morning of postoperative day 3 [19], and Hct_avg_ to the average of Hct_pre_ and Hct_post_. Patient blood volume (PBV) in liters was assessed according to the formula [17]**:** PBV = *k1* × height (m)^3^ + *k2* × weight (kg) + *k3,* where *k1* = 0.3669, *k2* = 0.03219, and *k3* = 0.6041 for men; and *k1* = 0.3561, *k2* = 0.03308, and *k3* = 0.1833 for women. If either reinfusion or allogeneic RBC transfusion was performed, TBL was calculated by adding the change in Hct and the volume transfused.

### Statistical analysis

Data were analyzed using SPSS 21.0 (IBM, Chicago, IL, USA). Unless otherwise stated, differences were assessed for significance using the chi-squared test or Fisher’s exact test for categorical data, or the *t* test for continuous data; the significance threshold for these analyses was *p* < 0.05. Multivariate logistic regression was performed to identify risk factors for red cell transfusion. Factors that may influence RBC transfusion risk based on clinical or biological knowledge were included as covariates in the regression. We first researched the previous literature and included 10 factors as follows: age, sex, BMI, ASA score, comorbidities, preoperative Hb level, DVT prophylaxis (1 = Yes), operation time, TXA use (1 = yes), hospital level (< 1000, 1000–2000 or > 2000 procedures/year) and surgeon level (<200, 200–300 or >300 procedures/year). Those variables identified in multivariate analysis as risk factors for RBC transfusion were controlled in analyses to determine TXA-associated outcomes.

As additional validation of the results obtained in the present study, our results were meta-analyzed together with relevant data from studies of TXA in hip fracture patients. The meta-analysis was conducted following the recommendations of Cochrane handbook and PRISMA guidelines. A systemic search of PubMed, EMBASE, and the Cochrane Library was performed to identify relevant studies in English published from inception to 1 August 2018. Additional studies were identified by reviewing the reference lists of other literatures. The following key words were used to search: (hip fracture, or hemiarthrolasty, and tranexamic acid). Only randomized controlled trials or cohort studies of TXA in hip fracture patients with adequate data on targeted outcomes and high quality were included. And the quality and risk of bias were assessed by two independent authors according to the criteria outlined in the Cochrane handbook for systemic reviews of interventions. The endpoints were rate of allogeneic RBC transfusion (according to a RBC transfusion protocol) and rate of DVT. Data were meta-analyzed using a fixed-effects model in Review Manager 5.2 (The Cochrane Collaboration, Oxford, UK). Results were reported as odd ratios (ORs) with associated 95% confidence intervals (95%CI).

## Results

### Patient demographics

**Fig. 1:**
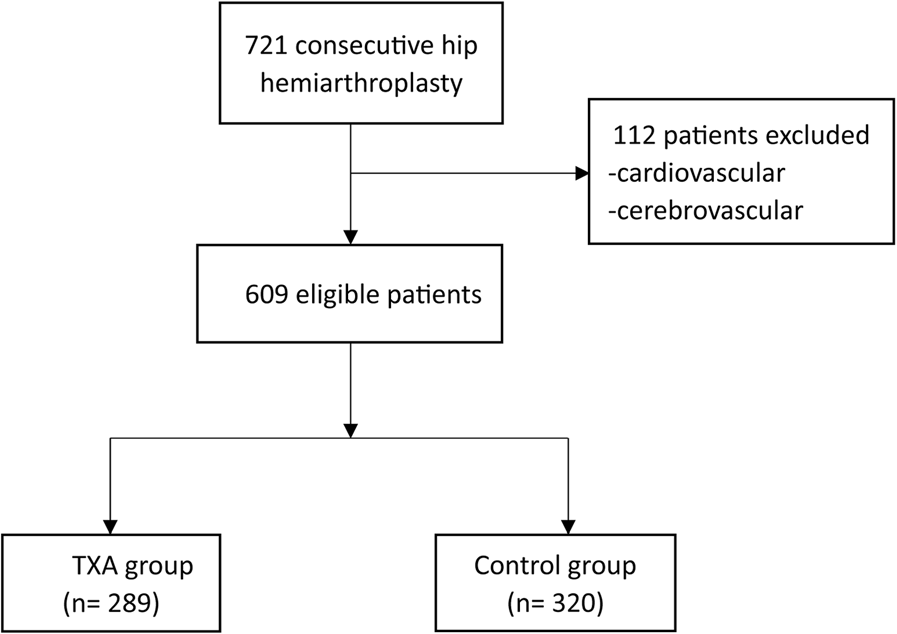
Flow diagram describing the number of patients included

During the study period, hemiarthroplasty was performed on 721 patients, of whom 112 were excluded because of cardio- or cerebrovascular problems (Fig. 1). The excluded patients underwent a blood management protocol similar to that of included patients but without TXA. The excluded patients did not differ significantly from the control patients in RBC transfusion requirements or adverse events (data not shown). The remaining 609 patients were included in the final analysis, comprising 289 who received prophylactic TXA and 320 who did not receive TXA (Table 1). The baseline characteristics were comparable between two groups. No patients were loss to follow up.

**Table 1:** Baseline characteristics of included patients

Variable	TXA group *N* = 289	Control group *N* = 320	P
Age, yr	84.41 ± 7.38	85.21 ± 8.27	0.210
Gender, F/M	198/91	214/106	0.666
Height, cm	162.72 ± 9.49	162.47 ± 7.65	0.724
Weight, kg	60.82 ± 12.90	58.60 ± 12.02	0.058
BMI, kg/m^2^	24.12 ± 2.49	22.11 ± 3.85	0.155
Anesthesia, n (%)			
General	129 (44.64)	114 (35.63)	0.057
CSE	79 (27.34)	110 (34.38)	
Spine	81 (28.03)	96 (30)	
ASA rating, n (%)			
I	63 (21.80)	86 (26.88)	0.230
II	116 (40.14)	107 (33.44)	
III	109 (37.72)	124 (38.75)	
IV	1 (0.34)	3 (0.93)	
Comorbidities, n (%)			
0	24 (8.30)	47 (14.69)	0.074
1	95 (32.87)	90 (28.13)	
2	112 (38.75)	121 (37.81)	
3	34 (11.76)	44 (13.75)	
≥ 4	24 (8.30)	18 (5.63)	
Pre Hb level	123.02 ± 14.65	120.74 ± 15.94	0.068
Pre Hct level	0.36 ± 0.04	0.35 ± 0.04	0.057
Anticoagulation therapy, n (%)			
Rivaroxaban	92 (31.83)	79 (24.69)	0.106
LMWH	181 (62.63)	216 (67.5)	
None	16 (5.54)	25 (7.81)	
Timing of initiation of VTE prophylaxis, n (%)			
Pre	159 (55.02)	180 (56.25)	0.068
≤ 12 h Post	105 (36.33)	94 (29.38)	
> 12 h Post	9 (3.11)	21 (6.56)	
None	16 (5.54)	25 (7.81)	
Pre ESA	156 (53.98)	183 (57.19)	0.426
Cementless/Cement, n/n	200/89	220/100	0.904
Operation time, min	88.19 ± 31.55	92.35 ± 49.16	0.220
Intra-Crystalloid, ml	785.12 ± 117.83	1063.42 ± 063.4	0.329
Intra-Colloid, ml	221.86 ± 195.85	312.00 ± 230.66	0.277
PBV, ml	3849.19 ± 639.74	37,771.80 ± 652.23	0.141
Hospital level			
<1000 procedures/year	108 (37.37)	109 (34.06)	0.693
1000–2000	109 (37.72)	128 (40.00)	
>2000	72 (24.91)	83 25.94()	
Surgeon level			
<200 procedures/ year	75 (25.95)	104 (32.50)	0.197
200–300	188 (65.05)	192 (60.00)	
>300	26 (9.00)	24 (7.50)	

Values are n (%) or mean ± SD

*BMI* body mass index; *CSE* combined spine-epidural anesthesia, *ASA* American Society of Anesthesiologists, *Hb* Hemoglobin, *Hct* hematocrit, *LMWH* low-molecular-weight heparin, *ESA* erythropoiesis-stimulating agents, *PBV* patient blood volume, *Post* postoperative, *Pre* preoperative

### Primary outcomes

Postoperative comparisons between the two groups are detailed in Table 2. The overall RBC transfusion rate was 16.75% (102 patients), with transfusions of at least 1 U of erythrocytes administered to 25 patients in the TXA group (8.65%) and to 77 in the control group (24.06%, *p* < 0.001). This corresponded to a significant relative risk reduction of 70% (OR 0.299, 95%CI 0.184 to 0.485). Total blood loss was 488.54 ± 296.24 ml in the TXA group, significantly lower than in the control group (589.13 ± 376.19 ml, p < 0.001). Mean Hb level was significantly higher in the TXA group on postoperative day 1 (111.70 ± 18.40 vs 107.29 ± 18.70 g/L, *p* = 0.008) and postoperative day 3 (108.16 ± 17.25 g/L vs 104.22 ± 15.16 g/L, *p* = 0.005). The TXA contained a significantly greater proportion of patients who began to ambulate within 24 h after surgery (37.02% vs 26.25%, *p* = 0.004), and TXA patients remained in the hospital for significantly fewer days (11.82 ± 4.39 vs 15.96 ± 7.30 days, *p* = 0.003).

**Table 2:** Primary outcomes

Variable	TXA group *N* = 289	Control group *N* = 320	P
Transfusion, n (%)			
Yes	25 (8.65)	77 (24.06)	< 0.001
No	264 (91.35)	243 (75.94)	
Hb on POD 1, g/l	111.70 ± 18.40	107.29 ± 18.70	0.008
Hb on POD 3, g/l	108.16 ± 17.25	104.22 ± 15.16	0.005
Hb drop on POD 1, g/l	18.26 ± 2.94	20.44 ± 3.25	0.411
Hb drop on POD 3, g/l	20.47 ± 2.88	34.43 ± 4.11	< 0.001
Total blood loss, ml	488.54 ± 296.24	589.13 ± 376.19	< 0.001
Intra blood loss, ml	230.17 ± 136.21	254.56 ± 161.38	0.046
Drain, n (%)			
Yes	221 (76.47)	236 (73.75)	0.439
No	68 (23.53)	84 (26.25)	
Drainage, ml	183.13 ± 111.44	218.27 ± 156.19	0.138
Ambulation time, n (%)			
≤ 24 h	107 (37.02)	84 (26.25)	0.004
> 24 h	182 (62.98)	236 (73.75)	
Length of stay, d	11.82 ± 4.39	15.96 ± 7.30	0.003

Values are n (%) or mean ± SD

*Hb* hemoglobin, *POD* postoperative day

Logistic regression identified preoperative Hb level, operation time, and DVT prophylaxis as risk factors for RBC transfusion in the TXA and control groups (Table 3). After adjusting for these covariates, TXA was associated with a relative reduction of RBC transfusion risk of 62% (OR 0.327, 95%CI 0.214 to 0.696).

**Table 3:** Multivariate analysis of the association between transfusion and perioperative risk factors in hip fracture surgery

Variable	B	S.E.	P^*^	OR	95%CI
TXA (1 = Yes)	−0. 953	0.301	0.002	0.386	0.214–0.696
Preoperative Hb	−0.019	0.008	0.018	0.982	0.967–0.997
DVT prophylaxis (1 = Yes)	1.140	0.486	0.035	2.801	1.186–7.179
Operation time	−0.024	0.005	0.001	1.087	1.081–1.098

^*^The following 10 factors were included in the analysis: age, sex, BMI, ASA score, comorbidities, preoperative Hb level, DVT prophylaxis, operation time, TXA use, hospital level (< 1000, 1000–2000 or > 2000 procedures/year) and surgeon level (< 200, 200–300 or > 300 procedures/year). Only variables with P < 0.05 are shown. *DVT* deep venous thrombosis, *Hb* hemoglobin, *TXA* tranexamic acid

### Complications

One patient in the TXA group and three patients in the control group developed DVT, which resolved in all cases after anticoagulation therapy was extended. No PE or mortality occurred during surgery or 3-month postoperative follow-up. The two groups did not differ significantly in rates of other adverse events (Table 4).

**Table 4:** Safety outcomes

Complication	TXA group	Control group	P
DVT	1 (0.35%)	3 (0.94%)	0.625
PE	0	0	–
Mortality	0	0	–
Pulmonary infection	2 (0.69%)	2 (0.63%)	1.000
Urinary tract infection	–	–	–
Cardiac infraction	–	–	–
Stroke	–	–	–
Congestive heart failure	–	–	–

*DVT* deep venous thrombosis, *PE* pulmonary embolism

## Discussion

This cohort study suggests that TXA is safe and effective for reducing blood loss, RBC transfusion rate, and length of hospital stay in geriatric hip fracture patients undergoing hemiarthroplasty. TXA in our study was associated with a 17.15% decrease in total blood loss, and a 64.05% decrease in RBC transfusion rate. Patients treated with TXA showed higher postoperative Hb levels, may have helped them recover from surgery.

Hemiarthroplasty is currently the most reliable treatment option for displaced subcapital hip fractures in geriatric patients with low function demands and no pre-existing acetabular wear [20]. Elderly hip fracture patients undergoing this procedure are more susceptible to blood loss and complications such as vascular thrombosis events than younger patients, mostly because of higher rates of comorbidities such as chronic pulmonary disease, congestive heart failure, diabetes, cerebrovascular disease, acute or previous myocardial infarction, cancer, peripheral vascular disease, and chronic renal failure [5, 21]. Despite the additional demands and risks in this subgroup of geriatric hip fracture patients, they have been quite underrepresented among the many studies of TXA safety and efficacy.

In addition, the large literature on TXA safety and efficacy has focused on elective lower total joint arthroplasty [10–13] but has neglected hip fracture surgery. Indeed, we were able to identify only nine studies [15, 22–29] analyzing the use of TXA in hip fracture surgery (Table 5). Previous studies included all types of hip fracture procedure, such as hemiarthroplasty, total hip arthroplasty, as well as dynamic hip screw and intramedullary nail procedures [15, 23–28]. TXA efficacy and safety may differ with type of hip fracture and surgery. Moreover, only two studies [22, 29] explored the safety and efficacy of TXA in older patients of high risk of complications, which included small sample of geriatric hip fracture patients undergoing hemiarthroplasty. Therefore in the present study we focused only on geriatric hip fracture patients older than 65 years with hemiarthroplasty.

**Table 5:** Studies of TXA in hip fracture procedures

Study	Design	Sample	Surgery type	TXA regimen	Conclusion
Zufferey 2010	RCT	110	Arthroplasty, DHS and IMN	15 mg/kg prior to surgery and 3 h later	TXA effective but not safe
Lee 2015	Cohort study	271	Hemiarthroplasty	1 g bolus preoperative	TXA safe and cost-effective
Sadeghi 2007	RCT	67	Internal fixation and hemiarthroplasty	15 mg/kg preoperative	TXA significantly reduces blood loss
Vijay 2013	RCT	90	Internal fixation and arthroplasty	10 mg/kg	TXA reduces blood loss and transfusion requirement
Baruah 2016	RCT	60	DHS	15 mg/kg preoperative	TXA safe and effective
Tengberg 2016	RCT	72	IMN	1 g preoperative and 3 g infusion for 24 h	TXA effective for reducing blood loss, but safety should be investigated further
Mohib 2015	RCT	100	–	15 mg/kg preoperative and 3 h later	TXA effective and safe
Emara 2014	RCT	60	Hemiarthroplasty	10 mg/kg prior to surgery and 5 mg/kg/h infusion until end of surgery, or 1.5 g for topical irrigation	Topical TXA safer than intravenous TXA
Watts 2017	RCT	138	Hemiarthroplasty or THA	2 dose of 15 mg/kg IV TXA before incision and at wound closure	TXA was safe to reduce blood loss with a tendency for decreased transfusion
Current study	Cohort study	609	Hemiarthroplasty	15 mg/kg prior to incision	TXA effective and safe

*DHS* dynamic hip screw, *IMN* intramedullary nail, *RCT* randomized controlled trial

**Fig. 2:**
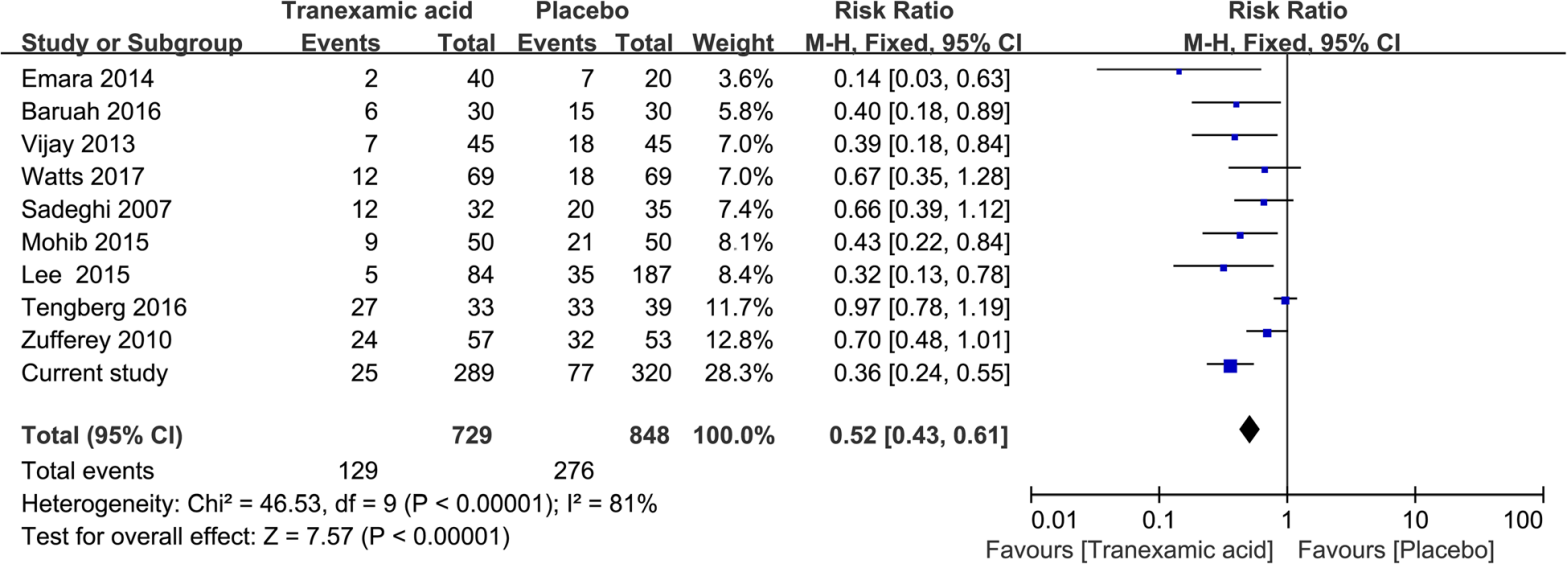
Meta-analysis of transfusion requirement

**Fig. 3:**
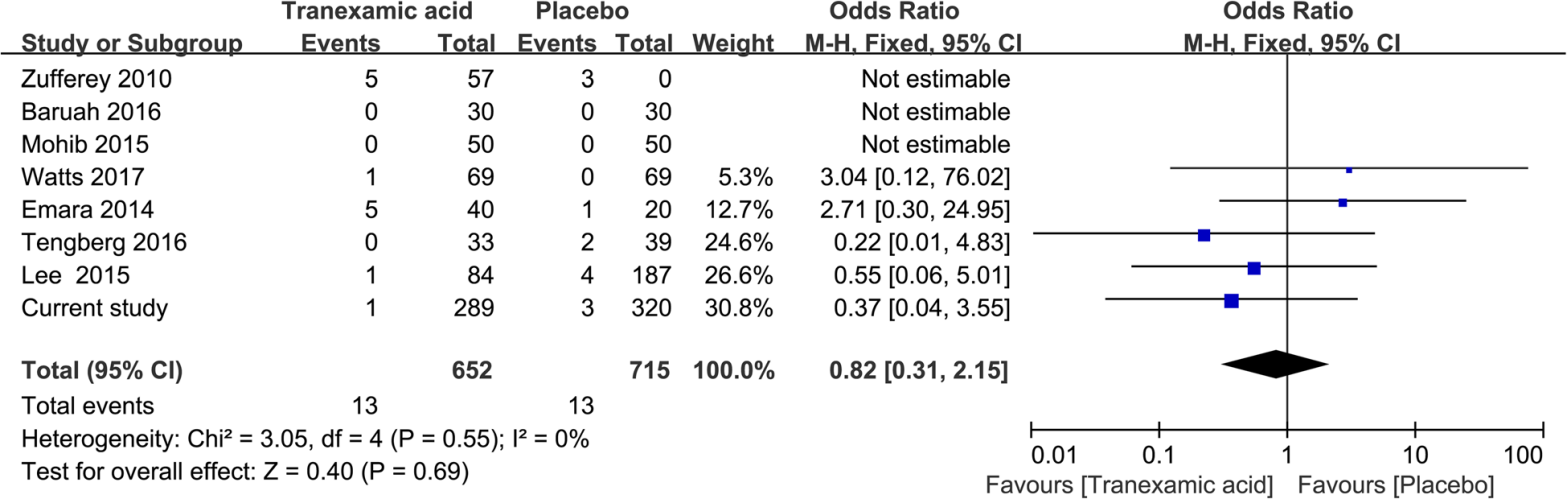
Meta-analysis of DVT

The first gap in the literature is the little sample (range from 60 to 271). For example, in the retrospective cohort study by Lee [22] involving 271 hip fracture patients undergoing hemiarthroplasty, TXA reduced the RBC transfusion requirement from 19 to 6%. However, only 84 patients (31%) in that study received TXA, highlighting the need for larger studies of TXA in hip fracture procedures. In our study, we included 609 geriatric hip fracture patients and pooled our data with data from other seven randomized controlled trials and one retrospective cohort studies [15, 22–29]. This meta-analysis indicated that TXA was associated with a significant reduction in the proportion of patients requiring allogeneic RBC transfusion (OR 0.52, 95%CI 0.43 to 0.61; Fig. 2), and that TXA was not associated with increased risk of DVT (OR 0.82, 95%CI 0.31 to 2.15, Fig. 3). The results in our study are consistent with those of a meta-analysis of data pooled from our data and from nine previous studies identified in our literature search. This also was the advantage of our study.

Another gap in the literature is studies on optimal TXA dosage, which in the study by Lee et al. [22] was 10 mg/kg, 15 mg/kg, or 1 g, usually delivered as a single bolus preoperatively or continuously by perfusion. In our study, we chose an intravenous dosage of 15 mg/kg injected prior to surgery; this was based on the previous work [16], in which authors found that a single bolus of 15 mg/kg was superior to 10 mg/kg for reducing RBC transfusion rate in patients undergoing total hip arthroplasty.

In our study, 25 patients in the TXA group (8.65%) received transfusions of at least 1 U of red blood cells, compared to 77 patients in the control group (24.06%). This corresponds to a RBC transfusion odds ratio of 0.299 (95%CI 0.184 to 0.485). This extent of RBC transfusion reduction with TXA is similar to that reported in other studies of TXA in hip fracture patients [23–28]. In a randomized controlled trial of hip fracture patients undergoing arthroplasty, dynamic hip screw surgery or intramedullary nail surgery [5], TXA lowered the RBC transfusion requirement (43% vs 60%) but the decrease was not statistically significant (*p* = 0.06). Therefore, those authors pooled their data with those of another trial of hip fracture patients [23] and found a significant reduction in RBC transfusion requirement with TXA (OR 0.47, 95%CI 0.26 to 0.85). This is consistent with our results. However, 112 patients underwent hemiarthroplasty without TXA were excluded because of cardio- or cerebrovascular conditions according to the inclusion and exclusion criteria. In order to clarify the potential selection bias, we performed a sensitivity analysis with these patients counted amongst the comparator group. And the results also indicated a similar hemostatic effect of TXA as before (RBC transfusion rate, 8.7% Vs 26.9%, OR = 0.258, *p* < 0.001).

Fast-track surgery, also called enhanced recovery after surgery, uses a multimodal approach to manage and alleviate the surgical stress response, with the goal of facilitating recovery [30]. All patients in our study received a fast-track protocol involving preoperative nutrition management, blood management, pain management, early mobilization, postoperative early oral intake and VTE prophylaxis. Our findings suggest that when TXA is combined with the fast-track protocol, it can improve the proportion of patients ambulating within 24 h after surgery, as well as shorten hospital stay. These benefits may reflect the lower blood loss and higher postoperative Hb level associated with TXA.

The most common safety concern with TXA is risk of VTE. Studies have shown trends toward greater incidence of postoperative vascular events with TXA in hip fracture patients (16% vs 6%, *p* = 0.10) [15] and higher 90-day mortality with TXA in patients with extracapsular fracture (27.2% vs 10.2%, *p* = 0.07) [25]. In the present study, we found no evidence that TXA was associated with greater risk of venous occlusive events or other adverse events, and our meta-analysis showed no increase in DVT risk. Nevertheless, because geriatric hip fracture patients are particularly vulnerable, TXA should be used with caution. Further larger studies are warranted to investigate TXA safety in different subgroups.

In our study, according to the inclusion and exclusion criteria, TXA was given to patients if deemed necessary by the surgeon, based on standard practices at the participating hospitals. This raises the possibility that different surgeons, or hospitals, were the factors that determined the differences between the study groups, which was the major potential flaw with our results. Therefore, in order to assess whether the potentially confounding factors of hospitals or surgeons may have contributed to the observed ability of TXA to reduce postoperative RBC transfusion rates, we conducted multivariate logistic regression **(**Table 3**).** Even after controlling for these potential confounders, TXA showed a significant hemostatic effect (OR 0.386, 95%CI 0.214 to 0.696) that was similar to the original results (OR 0.299, 95%CI 0.184 to 0.485) and to the meta-analysis of pooled data (OR 0.52, 95%CI 0.43 to 0.61). The results after controlling for potential confounders identified DVT prophylaxis as a RBC transfusion risk factor (OR 2.801, 95%CI 1.186 to 7.179). Perioperative anticoagulation therapy can reduce incidence of VTE but also increase risk of bleeding, although one study has suggested that preoperative anticoagulation therapy involving low-molecular-weight heparin may not increase intra- or postoperative bleeding [31].

In fact, further study is needed to address other limitations in the present work, which was not randomized and which focused only on hemiarthroplasty. The lack of randomization also meant that we could not ensure entirely comparable baseline characteristics between the TXA and control groups.

## Conclusion

Tranexamic acid is effective and safe for reducing blood loss and red blood cell transfusion requirements in fast-track hemiarthroplasty for geriatric hip fracture patients.

## Data Availability

The datasets used and analyzed during the current study are available from the corresponding author on reasonable request.

## Abbreviations

ASA: American Society of Anesthesiologists
BMI: Body mass index
CSE: Combined spinal-epidural anesthesia
DVT: Deep venous thrombosis
ERAS: Enhanced recovery after surgery
Hb: Hemoglobin
Hct: Hematocrit
MAP: Mean arterial pressure
PBV: Patient blood volume
PE: Pulmonary embolism
RBC: Red blood cell
SVV: Stroke volume variation
TBL: Total blood loss
TXA: Tranexamic acid
VTE: Venous thromboembolism
